# Occlusive retinal vasculitis associated with intravitreal Faricimab injections

**DOI:** 10.1186/s12348-024-00429-7

**Published:** 2024-09-27

**Authors:** Felix F. Reichel, Peter Kiraly, Roopa Vemala, Stella Hornby, Samantha R. De Silva, M. Dominik Fischer

**Affiliations:** 1 Centre for Ophthalmology, University Eye Hospital, University Hospital Tübingen, Tübingen, Germany; 2 Oxford Eye Hospital, Oxford University Hospitals NHS Foundation Trust, Oxford, UK

**Keywords:** Faricimab, Vabysmo, Occlusive vasculitis, Diabetic retinopathy

## Abstract

**Purpose:**

We describe a case of occlusive vasculitis associated with intravitreal Faricimab (Vabysmo) injections.

**Methods:**

A retrospective case report.

**Results:**

A 52-year old man treated with monthly Faricimab injections for diabetic macula oedema presented with sudden reduced vision, new retinal hemorrhages, significant retinal vascular occlusions and ischemia. After screening for differential diagnoses was unremarkable, the patient was treated with oral and intravitreal steroid therapy under which the occlusive vasculitis was stabilized.

**Conclusion:**

Occlusive vasculitis, though rare, is a potential complication of Faricimab therapy. Comprehensive reporting and large-scale analyses are essential to better understand and manage this adverse event.

## Introduction

Faricimab, marketed as Vabysmo, is a bispecific antibody designed to bind and neutralize angiopoietin (Ang)-2 and VEGF-A with high specificity and potency [1]. The phase III safety and efficacy trials YOSEMITE/RHINE demonstrated non-inferiority to Aflibercept (Eylea) for use in age-related macular degeneration (AMD) and diabetic macula oedema which led to approval by the U.S. Food and Drug administration and European Medicines Agency in 2022. Additionally, the trials suggested a reduction of treatment burden with Faricimab, which has been supported by real-world data [2, 3]. With regards to safety, a comparable risk profile to aflibercept was shown in the Phase III trials. However, although the RHINE and YOSEMITE did not report any cases of vasculitis or retinal artery occlusions, Genentech recently updated the Faricimab label to include the risk of retinal vasculitis, with an estimated rate of occlusive retinal vasculitis of 0.06 per 10,000 injections [4]. In comparison to other anti-VEGF drugs, this rate is lower than the reported rate for Brolucizumab (Beovu) injections (2.35 per 10,000) but slightly higher than Aflibercept, where intraocular inflammation with retinal artery occlusion or retinal/ocular vasculitis was reported at a rate of approximately 1 per 6 million Aflibercept vials sold (< 0.00002%), with most cases associated with endophthalmitis [5, 6].

## Case report

A 52-year-old male patient with type II diabetes and bilateral non-proliferative diabetic retinopathy complicated by diabetic macula oedema presented with a 4-day history of suddenly reduced and patchy vision in his right eye. This occurred 35 days after the last intravitreal injection of Faricimab (Vabysmo). His visual acuity in the right eye had declined from 20/25 at the time of the last injection to 20/63, while the visual acuity in his left eye remained stable at 20/32. Over the course of his treatment for diabetic macular oedema, both eyes had received six Faricimab injections, with the right eye treated over 8 months and the left over 7 months, resulting in the complete resolution of macular oedema in both eyes. The patient had been treatment-naïve before Faricimab injections were initiated. The patient's HbA1c was 7.9% (63 mmol/mol).

**Fig. 1:**
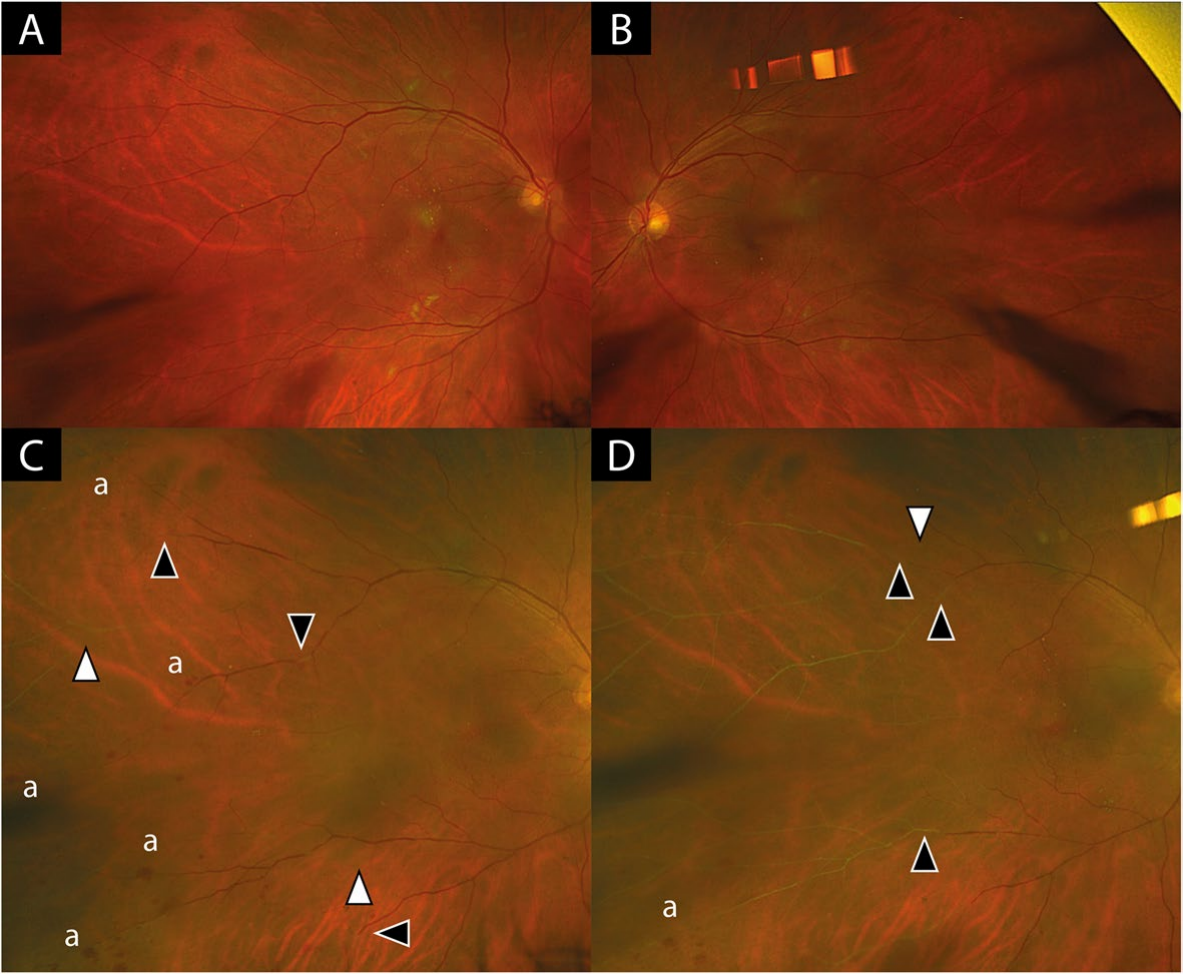
Widefield-fundus imaging (Optomap P200) of right (**A**, **C**, **D**) and left eye (**B**). **A** and  **B** shows fundus imaging at last follow-up 3 months before patient became symptomatic with findings of mild to moderate non-proliferative diabetic retinopathy but no signs of vascular occlusion. **C** shows fundus image of right eye of patient at presentation with new blot hemorrhages (a) in the temporal periphery, severe attenuation of arterial vessels (white arrow heads) and veins (black arrow heads). **D**: shows progression of vessel occlusion 4 weeks after initiation of treatment. Arrow heads indicating progression of vessel occlusions compared to **C**

**Fig. 2:**
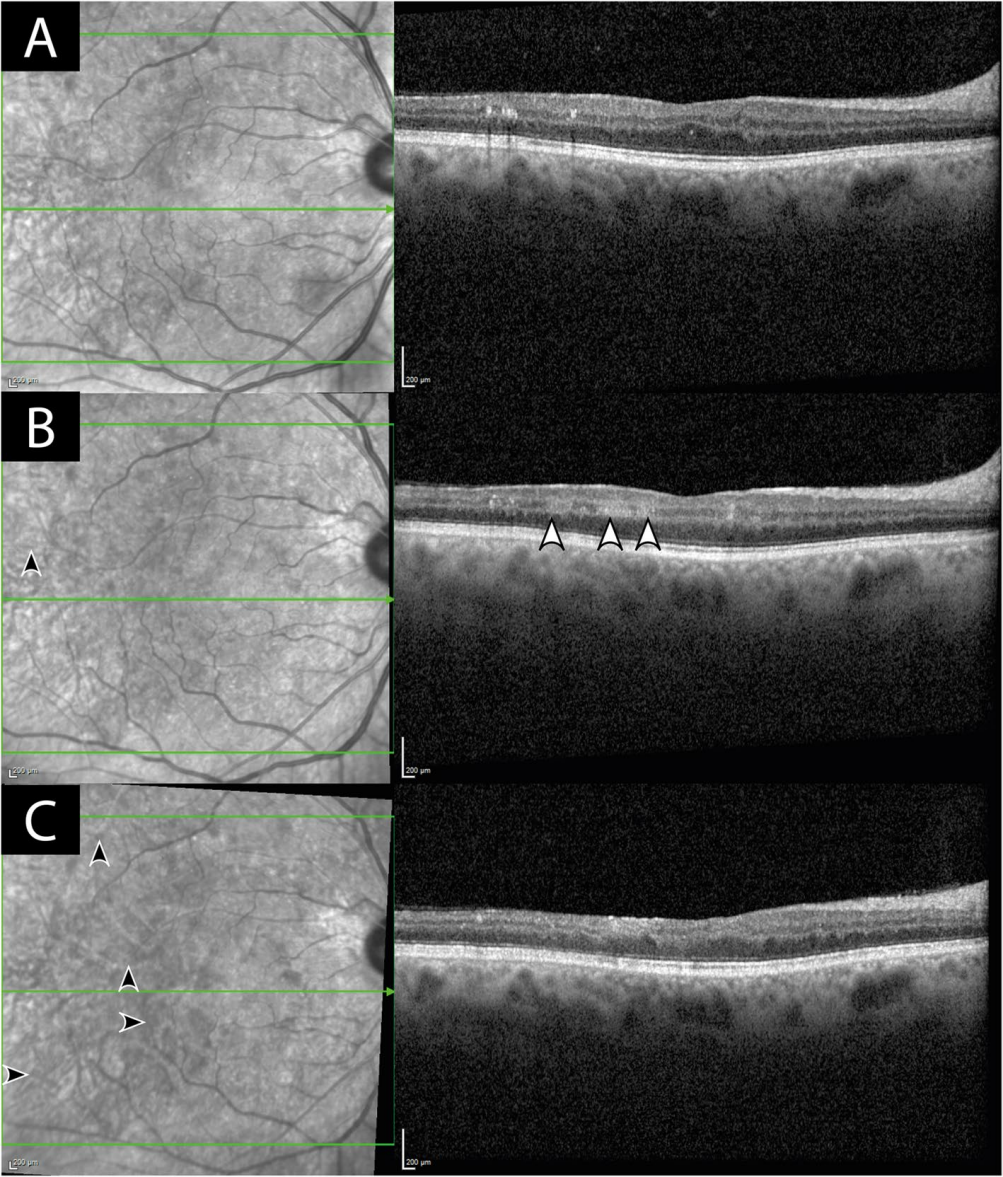
Infrared reflectance (IR) images with optical coherence tomography (OCT) scans (Spectralis Heidelberg) of right eye at 3 months before the patient became symptomatic (**A**), at initial presentation (**B**) and 4 weeks later (**C**). White arrow heads denote hyperreflectivity consistent with paracentral acute middle maculopathy (PAMM) as a sign of ischemia in the intermediate and deep retinal capillary plexuses. In the infrared images on the left side dark arrow heads mark the progression of vessel attenuation compared to the previous image

**Fig. 3:**
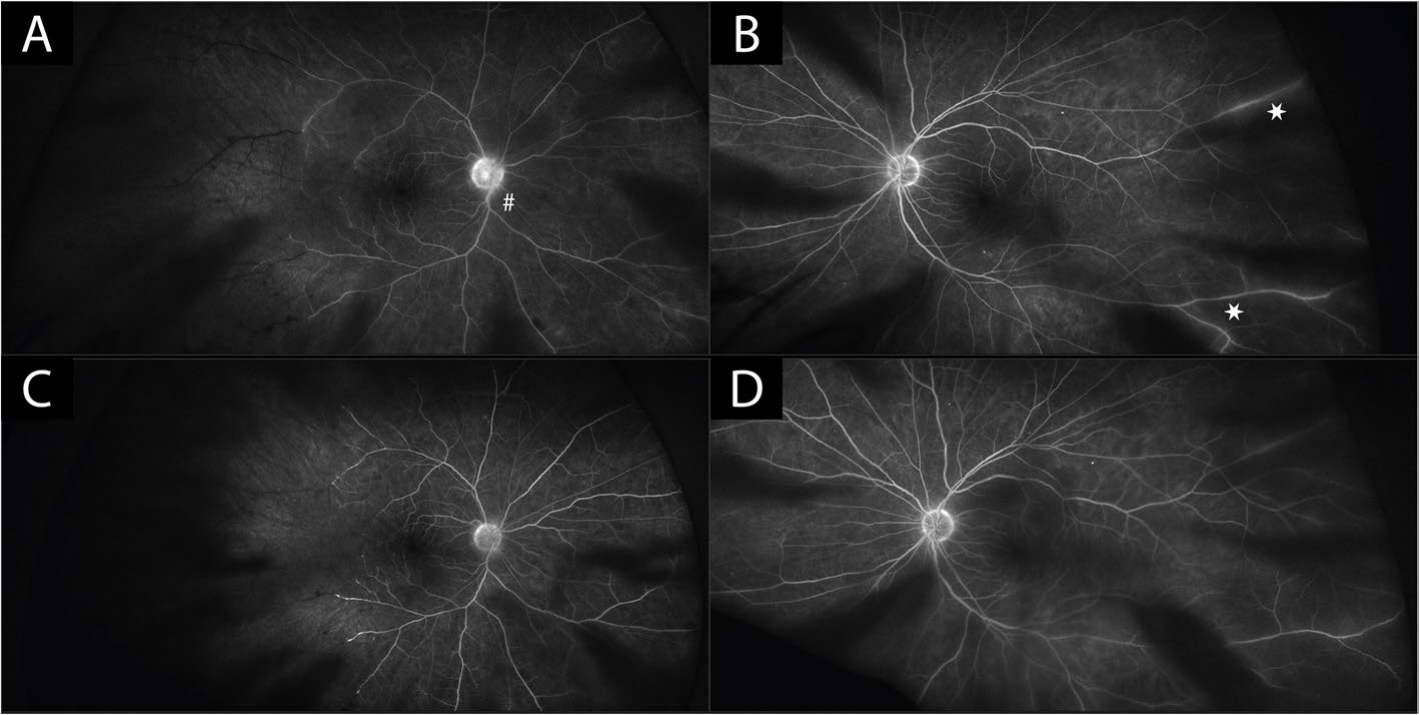
Fluorescein angiography in the right (**A**; at 6 min 39 s) and the left (**B**; at 3 min 53 s) eye at initial presentation confirming significant ischemia in the right eye, staining of optic discs (#), and fluorescein leakage of retinal veins especially in the left eye (asterisks). Fluorescein angiography 4 weeks after treatment initiation (right—**C**; at 4 min 6 s and left—**D**; at 4 min 24 s) shows slight progression of vascular occlusions in the right eye and no occlusions in the left eye

Ophthalmic examination showed no cells in the anterior chamber or vitreous in either eye. Fundus examination of the right eye revealed new blot hemorrhages in the temporal periphery and attenuation of both peripheral and central retinal arteries and veins, as seen on Optos widefield-imaging (Optomap P200; Optos plc, Dunfermline, UK) in Fig. 1 and on fluorescein angiography in Fig. 3, A-B. Optical coherence tomography (OCT) imaging of the macula revealed signs consistent with paracentral acute middle maculopathy in the right eye (Fig. 2). The left eye showed mild fluorescein leakage from peripheral veins but was otherwise unremarkable.

The patient had no history of vascular occlusive disease, a normal coagulation profile, normal blood pressure and no clinical or laboratory signs of giant cell arteritis. Comprehensive retinal vasculitis screening results including chest X-ray, Quantiferon test, serology for Syphilis, Human Immunodeficiency Virus, Hepatitis B/C Virus, cryoglobulins, rheumatoid factor, angiotensin-converting enzyme, antineutrophilic cytoplasmic antibody and antinuclear antibody were all unremarkable. CT-Angiography of the head and neck revealed conventional anatomy, normal opacification and no stenosis of aortic arch, extracranial neck vessels, major intracranial vessels and the ophthalmic arteries bilaterally. Given the diagnosis of an occlusive vasculitis, the patient was treated with oral Prednisolone at 1 mg/kg bodyweight, tapering down at weekly intervals. Despite treatment, after 4 weeks the vascular occlusions had further progressed towards the centre. However, the oral prednisolone therapy was still tapered due to worsening blood sugar levels and instead the right eye received an intravitreal injection of a Dexamethasone implant (Ozurdex). Three weeks later the area of vascular occlusion had stabilized (Fig. 3, C-D) and visual acuity slightly improved to a final visual acuity of 20/40 in the right eye and 20/25 in the left eye.

## Discussion

Herein, we report a case of occlusive vasculitis associated with Faricimab injections in a patient with diabetic retinopathy. A causative relationship with Faricimab was suspected given the time course of onset, the known (albeit rare) incidence of vasculitis associated with Faricimab and the absence of other causes. While retinal ischemia can occur in diabetic retinopathy, the progression of retinal non-perfusion is typically slow, especially in the presence of ongoing anti-VEGF therapy [7]. Although no fluorescein angiography images were available before the onset of symptoms, widefield Optos images clearly demonstrated normal peripheral vessels. Also, there are similarities when comparing findings in this patient to those with occlusive vasculitis after Brolucizumab injections. In Brolucizumab associated cases of occlusive vasculitis, the onset was often not immediately after the injection but occurred between 1 week and over 12 months after the first injection [5]. This supported the hypothesis of a delayed-type hypersensitivity reaction as the origin [8]. It was speculated that anti-drug antibodies (ADA) against a non-native species of Brolucizumab, which form upon prolonged incubation at body temperature in the vitreous, led to immune complex formation as mediators of the hypersensitivity reaction and trigger of inflammation and platelet aggregation [9]. Although a completely different mechanism could be at play in the case of Faricimab associated vasculitis, experience from Brolucizumab shows that a time interval of several months between the first injection and the onset of an occlusive vasculitis does not exclude a causal relationship. Notably, the onset within 5 weeks of the last injection, as well as the presentation of an occlusive vasculitis without signs of intraocular inflammation is consistent with that found in association with Broluzicumab [5, 10]. A relationship between the occlusive events in the patient reported here with faricimab therefore seems plausible. To date, only two case reports of an occlusive vasculitis associated with Faricimab injections has been published. In one case an elderly woman with wet AMD developed intraocular inflammation with associated occlusive vasculitis after a single Faricimab injection following a switch from Aflibercept [11]. In the other case a 72-year old man treated for polypoidal choroidal vasculopathy developed occlusive vasculitis two weeks after the second Faricimab injection [12]. Cases of sterile intraocular inflammation with presumed association to Faricimab but without signs of vasculitis have also been reported. A case series from one institution described 3 patients with severe sterile intraocular inflammation within a 1-month time frame after receiving Faricimab for wet age-related macular degeneration [13]. Another report described a case of intraocular inflammation associated with significantly increased intraocular pressure in a patient with diabetic macula oedema 4 weeks after the third injection [14].

These reports suggest that Faricimab can potentially elicit sterile inflammation and occlusive vasculitis. While case reports underscore the need for ongoing vigilance to detect this rare complication, large-scale studies are essential to accurately determine its frequency. Such studies will potentially aid clinicians and patients in making informed decisions regarding the risks, especially in comparison to other anti-VEGF therapies.

## Conclusion

Occlusive vasculitis is a rare complication of Faricimab. Large-scale database analyses will be necessary to determine the frequency of occlusive vasculitis, with or without intraocular inflammation, in patients receiving Faricimab therapy. Until then, signs of vascular occlusive events in patients undergoing this therapy should be carefully evaluated and reported as potential adverse events.

## Data Availability

The data supporting the findings of this case report are included within the manuscript. Due to the nature of this research, the raw data and clinical images cannot be shared publicly to protect patient privacy. However, additional details and clarifications can be provided upon reasonable request to the corresponding author, subject to institutional and ethical guidelines.

## Abbreviations

VEGF: Vascular endothelial growth factor
AMD: Age related macular degeneration
OCT: Optical coherence tomography
CT: Computed tomography
PAMM: Paracentral acute middle maculopathy
ADA: Anti-drug antibodies
